# Parental Knowledge and Attitudes Towards Milk Fluoridation for Dental Caries Prevention in the United Kingdom

**DOI:** 10.7759/cureus.90082

**Published:** 2025-08-14

**Authors:** Joyce E Idomeh, Kennedy O Obohwemu, Gordon M Yakpir, Friday I Ndioho, Osinubi Olusunmola, Aduke E Ipingbemi, Rupali Chauhan, Divya Motupalli

**Affiliations:** 1 College of Education, Psychology and Social Work, Flinders University, Adelaide, AUS; 2 Faculty of Health, Wellbeing and Social Care, Oxford Brookes University, Birmingham, GBR; 3 Department of Health Professions, Manchester Metropolitan University, Manchester, GBR; 4 Department of Health, East Kent Colleges Group, Ashford Kent, GBR; 5 Faculty of Health, Wellbeing and Social Care, Oxford Brookes University, Manchester, GBR

**Keywords:** attitude of parents, deprived households, knowledge, school-aged children, teeth

## Abstract

Background: A significant proportion of school-aged children in some developed nations suffer from dental caries, which causes discomfort, pain, and reduced school attendance for children. Associated risk factors include socioeconomic status, diet, and access to dental care. The main objective of this study was to evaluate the knowledge and attitudes of parents of young children aged 4-11 regarding milk fluoridation as a caries preventive measure.

Methods: A quantitative cross-sectional research method was adopted for this study; we recruited participants conveniently with an online validated questionnaire that examined the general knowledge and attitudes the respondents had towards milk fluoridation. Data were analysed using descriptive, univariate, and multivariate regression.

Results: Data analysis revealed poor knowledge and general negativity towards milk fluoridation in Gateshead. Over half (51.3%) of the respondents were not sure of the benefits of fluoridation and what it stood for. Despite the poor knowledge of fluoridation, 61.6% of the parents agreed that they would allow their children to be given fluoridated milk if given the opportunity. A significant relationship was observed between the level of education and monthly income. It was observed that secondary-level education increased the likelihood (p = 0.048) seven times (B = 7.000) of the respondents having adequate knowledge of fluoridation.

Conclusions: Undoubtedly, there remains limited information on general knowledge about milk fluoridation in Gateshead. It is, therefore, recommended that educational interventions and socioeconomic development be implemented before any community-level milk fluoridation program in Gateshead.

## Introduction

Dental caries is a complex disease that occurs because of the contact between probiotic bacteria and carbohydrate food substances with the teeth's enamel or the surface of the mouth [1]. Dental caries is still one of the most important preventable oral health problems that affect the general health of populations worldwide [2]. It is a painful condition that affects the health of children, who may develop pain, inability to concentrate in school, and absence from school [3,4].

In developed nations, more than 85% of school children are affected by dental caries [5], including vulnerable populations, with a worse quality of life. Such factors as health inequalities, economic status, diet, and inadequate oral hygiene practices at home are some risk factors that increase the occurrence of dental caries [6]. These include dietary behaviours, oral hygiene practices, knowledge, and beliefs, which are important in the prevention and control of the disease [7].

Fluoride programs have been in effect for decades worldwide [8,9]. However, there are still debates on the possible adverse health effects of fluoridation interventions [10]. In the United Kingdom, for example, where the application of fluoride has been in place for more than 50 years, the debate on the effectiveness of fluoride still exists [11]. Thus, alternative preventive measures such as milk fluoridation are considered to be more effective in meeting the oral health needs of children in different socioeconomic populations [12]. Furthermore, milk is a source of nutrients that help in growth [6,7].

Milk fluoridation has been effective in several countries, including Hungary, Israel, Bulgaria, the USA, China, Thailand, Sweden, Russia, and some parts of the United Kingdom including Scotland [13]. The concept of fluoridation of milk was also learned from previous studies on fluoridated milk and the creation of ‘The Borrow Dental Milk Foundation’, which paved the way for further studies on the effectiveness of putting fluoride in milk instead of water as a caries prevention strategy [13]. A great benefit of this approach is that the fluoride can be delivered in a targeted, safe, and cost-effective manner through milk as compared to other costly interventions for dental caries [13].

To the best of our knowledge, there is very limited information on milk fluoridation in the UK, especially in Northeast England. This study will determine the perceptions and understanding among parents of young children on the subject of milk fluoridation in the prevention of dental caries.

## Materials and methods

Study design and setting

Study Period and Population

This was a cross-sectional study. The study population included guardians of children aged 4-11 residing in Dunston and Teams Ward, Gateshead, located in the metropolitan county of Tyne and Wear, Northeast England. Dunston and Teams Ward, in the inner West of Gateshead, has a population of 1,895 children aged 0-19 years. The area is ranked among the 10% most deprived in England, with a life expectancy of 78 years [2].

Sampling Strategy

A convenience sampling method was employed for participant recruitment. The inclusion criteria were parents aged 18 and above who had resided in Dunston and Teams Ward for at least three months and had children aged 4-11. Participants were required to provide voluntary consent after being informed about the study's purpose, content, and confidentiality.

Data Collection Procedure

The survey instrument was an online questionnaire consisting of 34 questions divided into four sections: Section A: Sociodemographics of parents and children; Section B: Questions about the child’s oral health; Section C: Parents' attitudes towards their children's oral health over the past three months, with subsections on oral symptoms, functional limitations, and emotional and social well-being; and Section D: Questions around knowledge and attitudes toward milk fluoridation.

Sample Size

The sample size was determined [14] and amounted to 406 participants with specific consideration to the critical and standard value at a 95% confidence interval, expected prevalence, and participants’ withdrawal, as indicated below:

$N = \frac{(Z_{1 - &alpha;/2})^{2}(p)(q)}{d^{2}}$

where N = the desired sample size, (Z_1 - α/2_)^2^ = critical and standard value at a 95% confidence interval, p = expected prevalence = 0.4, q = 1 - p = 1 - 0.4, d = margin of error, N = (1.96)^2^(0.4)(0.6)(0.05)/(0.05)^2^, N = 368.79 + 37 (considering participants’ withdrawal), N = 406 participants.

Statistical analysis

Data were analysed using Microsoft Excel (Microsoft Corp., Redmond, WA, US) and SPSS (SPSS Inc., Chicago, IL, US) for descriptive and inferential statistics. Descriptive statistics provided demographic information, while inferential statistics assessed relationships between dependent and independent variables. Independent variables included the parents' age, sex, nationality, and social status, while dependent variables included parents’ knowledge and attitudes towards milk fluoridation. The analysis used univariate and multivariate regression methods. A p-value of less than 0.05 was considered statistically significant.

Ethical consideration

Ethics approval was granted by the University of Sunderland's ethics committee. Data collection followed the Data Protection Act of 2018 [7].

Risk assessment

The risks for participants were minimal, as surveys were completed online at their convenience.

## Results

Basic characteristics of participants

The demographic characteristics of the parents are presented in Table 1. Data were collected from 85 parents for this study, with female respondents (N = 71, 83.5%) five times higher than male respondents (N = 14, 16.5%). A large number of the respondents (N = 66, 90%) were between 31 and 45 years old, and 5.9% (N = 5) were over 45 years old. For racial background, 80% (N = 68) of the participants were Blacks, 14.1% (N = 12) were White, 3.5% (N = 3) were Asians, and 2.5% (N = 2) were other mixed ethnic groups; 68.1% (N = 58) of the respondents were highly educated, and at least one parent had a post-tertiary education while 22.4% (N = 19) had at least a parent having tertiary education. Only 7.1% (N = 6) of the parents had secondary education while 1.2% (N = 1) had primary education. The largest group of respondents was public servants (N = 36, 42.9%), followed closely by private employees (N = 33, 39.2%), self-employed (N = 10, 11.9%), and those not employed (N = 5, 6.0%). More than a quarter of the respondents (N = 30, 35.7%) earned between £1,501 and £2,500 monthly, 28.6% (N = 24) earned between £501 and £1,500, 23.8% (N = 20) earned over £2,501, and only 9.5% (N = 8) of respondents were earning below £500 while 2.4% (N = 2) had no monthly earnings.

**Table 1:** Summary analysis of parents’ characteristics Data are represented as frequency (N) and percentage (%). Statistical significance is considered at p < 0.05.

Variables	Frequency	Percentage (%)
Parents' sex		
Female	71	16.5
Male	14	83.5
Parents' nationality		
Black	68	80.0
Asian or Asian British	3	3.5
Mixed or multiple ethnic groups	2	2.5
White	12	14.1
Parents' educational level		
None	1	1.2
At least one parent with primary education	1	1.2
At least one parent with secondary education	6	7.1
At least one parent with tertiary education	19	22.4
At least one parent with post-tertiary education	58	68.1
Parents' occupation		
None	5	6.0
Self-employed	10	11.9
Public servant	36	42.9
Private employment	33	39.2
Parents' age		
Years	14	16.5
31-45 years	66	77.6
46 years	5	5.9
Parents’ monthly income		
None	2	2.4
£500	8	9.5
£501-£1,500	24	28.6
£1,501-£2,500	30	35.7
£2,501	20	23.8

Children’s demography and oral health

The characteristics of the children are reported in Table 2. A total of 85 children were reported by the respondents and included males (N = 43, 50.6%) and females (N = 42, 49.4%). The majority of the children were aged 5-7 years (35.7%, N = 30) and between the ages of 10 and 11 years (N = 8, 9.5%).

**Table 2:** Summary of children’s characteristics Data are represented as frequency (N) and percentage (%). Statistical significance is considered at p < 0.05.

Variables	Frequency	Percentage (%)
Child’s/ward’s sex		
Female	42	49.4
Male	43	50.6
Child’s/ward’s age		
4-5 years	26	31.0
5-7 years	30	35.7
8-10 years	20	23.8
10-11 years	8	9.5
Child’s/ward’s school year		
Reception	21	25.6
Years 1-3	33	40.2
Years 4-6	23	28.0
Years 7-9	2	2.4
9	3	3.7

More than half of the children (N = 45; 55.6%) brushed their teeth once every day; the others brushed theirs twice daily and more than once weekly. The frequency of snacking on sweet things was observed to be more than once daily (N = 31, 38.3%), more than twice daily (N = 22, 27.2%), once a week (N = 11, 13.6%), or more than once weekly (N = 17, 21%) (Table 3).

**Table 3:** Summary of the child’s (ward’s) oral health activities Data are represented as frequency (N) and percentage (%). Statistical significance is considered at p < 0.05.

Variables	Frequency	Percentage (%)
Toothbrushing frequency		
Once every day	45	55.6
At least twice a day	32	39.5
More than once weekly	4	4.9
Frequency of snacking on sweet things		
Once every day	31	38.3
At least twice a day	22	27.2
Once a week	11	13.6
More than once weekly	17	21.0
Dentist visit frequency		
Once every day	7	8.9
At least twice a day	13	16.5
Once a week	21	26.6
More than once weekly	10	12.7
Never	28	35.4

Furthermore, most of the children never had food caught up in between their teeth (N = 42, 50.6%) or felt pain in their teeth (N = 66, 81.5%), never had difficulty eating hot or cold foods (N = 69, 90.8%), never had restricted diets (N = 66, 88.0%), or never even breathe through their mouths (N = 53, 68.8%) (Table 4).

**Table 4:** Summary analysis of the child’s (ward’s) oral symptoms and functional limitations Data are represented as frequency (N) and percentage (%). Statistical significance is considered at p < 0.05.

Variables	Frequency	Percentage (%)
Incidence of food caught in between teeth		
Never	42	50.6
Every day	15	18.1
Once weekly	18	21.7
Twice weekly	8	9.6
Incidence of pain in the teeth		
Never	66	81.5
Every day	0	0.0
Once weekly	11	13.6
Twice weekly	4	4.9
Difficulty drinking/eating hot/cold foods		
Never	69	90.8
Every day	4	5.3
Once or twice weekly	1	1.3
Once a month	2	2.6
Breathing through mouth		
Never	53	68.8
Every day	8	10.4
Once or twice weekly	5	6.5
Once a month	11	14.3
Restricted diet		
Never	66	88.0
Every day	6	8.0
Once or twice weekly	1	1.3
Once a month	2	2.7

Moreover, there seemed to be a balanced emotional and social well-being for the children as illustrated in Table 5. More than half of the children were less worried about being attractive (N = 69, 84.1%), never worried about being embarrassed (N = 61, 77.2%), never anxious (N = 53, 69.7%), always wanting to speak in class (N = 61, 78.2%) and talk with other children (N = 61, 78.2%), and never felt left out (N = 58, 76.3%).

**Table 5:** Summary analysis of a child’s emotional and social well-being Data are represented as frequency (N) and percentage (%). Statistical significance is considered at p < 0.05.

Variables	Frequency	Percentage (%)
Worried about being less attractive than others		
Never	69	84.1
Once a day	3	3.7
At least twice a day	0	0.0
Once weekly	8	9.8
More than once a week	2	2.4
Worried about being embarrassed		
Never	61	77.2
Once a day	5	6.3
At least twice a day	1	1.3
Once weekly	8	10.1
More than once a week	4	5.1
Anxious		
Never	53	69.7
Once a day	4	5.3
At least twice a day	2	2.6
Once weekly	12	15.8
More than once a week	5	6.6
Not wanting to speak aloud in class		
Never	61	78.2
Once a day	5	6.4
At least twice a day	1	1.3
Once weekly	4	5.1
More than once a week	7	9.0
Not wanting to talk to other children		
Never	61	78.2
Once a day	5	6.4
At least twice a day	3	3.8
Once weekly	8	10.3
More than once a week	1	1.3
Left out by other children		
Never	58	76.3
Once a day	7	9.2
At least twice a day	1	1.3
Once weekly	7	9.2
More than once a week	3	3.9

Parents’ knowledge, attitude, and practice towards fluoridation

The summary analysis of parents’ knowledge and awareness of fluoridation is illustrated in Tables 6, 7. Over half of the respondents were not sure of what fluoridation was (N = 39, 51.3%) or its benefit (N = 42, 55.3%).

**Table 6:** Summary analysis of parents’/guardians’ knowledge of fluoridation Data are represented as frequency (N) and percentage (%). Statistical significance is considered at p < 0.05.

Variables	Frequency	Percentage (%)
Adequate knowledge		
Agree	18	23.7
Disagree	19	25.0
Not sure	39	51.3
Most effective dental caries preventive methods		
Water fluoridation	14	18.4
Milk fluoridation	11	14.5
Water/milk fluoridation/professionally applied topical fluoride	10	13.2
Fluoride toothpaste	31	40.8
Fluoride supplements	2	2.6
Fluoride rinses	2	2.6
Caries cannot be prevented	6	7.9
Milk fluoridation benefits the oral health of children		
Agree	30	39.5
Disagree	4	5.3
Not sure	42	55.3

**Table 7:** Summary analysis of fluoridization status at Gateshead Data are represented as frequency (N) and percentage (%). Statistical significance is considered at p < 0.05.

Variables	Frequency	Percentage (%)
Knowledge of fluoridization		
Yes	9	12.2
No	11	14.9
Not sure	54	73.0
Milk should be optimally fluoridated in Gateshead		
Agree	27	36.5
Disagree	5	6.8
Not sure	42	56.8
Not in support of milk fluoridation because:		
It is hazardous to the environment	4	5.0
It violates personal freedom of the population	7	8.8
It has adverse effects on human general health	6	7.5
It is associated with allergy in some people	12	15.0
It is associated with adverse effects on human bones	5	6.3
It can cause dental fluorosis	12	15.0
Can cause cancer in humans	7	8.8
Has neurological side effects in humans	6	7.5
It is economically expensive	21	26.3

Table 7 shows poor knowledge of fluoridation, with 73% (N = 54) of the respondents not sure what fluoridation was and 56.8% (N = 42) also not sure if fluoridation is needed to be implemented in Gateshead to mitigate dental caries. Despite the poor knowledge of fluoridation, 61.6% (N = 45) of parents agreed to allow their children to be given fluoridated milk even if most of them (N = 60, 81.1%) do not give fluoridation milk presently to their children. Additionally, 51.3% (N = 39) were not so sure if fluoridation prevents dental caries (Table 8).

**Table 8:** Summary analysis of parents’ attitudes and practices towards fluoridation Data are represented as frequency (N) and percentage (%). Statistical significance is considered at p < 0.05.

Variables	Frequency	Percentage (%)
Fluoridation prevents dental caries		
Agree	18	23.7
Disagree	19	25.0
Not sure	39	51.3
Fluoridated milk preferable to other forms of fluoridation		
Agree	14	18.4
Disagree	11	14.5
Not sure	10	13.1
Do you give your children fluoridated milk?		
No	60	81.1
Yes	14	18.9
Will you allow your children to be given fluoridated milk?		
No	28	38.4
Yes	45	61.6

Confounding factors for milk fluoridation

Univariate linear regression analyses examined the correlation between the parents’ social status and their knowledge and practices related to dental caries (Tables 9-11). The only significant relationship was observed between the social status variables of no formal education (p = 0.062) and no monthly income (p = 0.013) with the knowledge of milk fluoridation (Table 12).

**Table 9:** Summary analysis of the association between parents Data represented as frequency (N) and percentages (%). Statistical association tested using Chi square or Fisher's test (superscript f where applicable). p < 0.05 is considered significant.

	Toothbrushing frequency					Snacking on sweet things					Dentist visitation						
Variables	Once every day	At least twice a day	More than once weekly	Chi square	p-value	Once every day	At least twice a day	More than once weekly	Chi square	p- value	Once every day	At least twice a day	Once a week	More than once weekly	Never	Chi square	p-value
Parents’ educational level																	
None	0 (0.0)	1 (100.0)	0 (0.0)	4.524	0.853^f^	1 (100.0)	0 (0.0)	0 (0.0)	9.623	0.558^f^	0 (0.0)	0 (0.0)	1 (100.0)	0 (0.0)	0 (0.0)	8.827	0.881^f^
Primary	1 (100.0)	0 (0.0)	0 (0.0)			1 (100.0)	0 (0.0)	0 (0.0)			0 (0.0)	0 (0.0)	1 (100.0)	0 (0.0)	0 (0.0)		
Secondary	4 (66.7)	2 (33.3)	0 (0.0)			3 (50.0)	2 (33.3)	1 (16.7)			1 (16.7)	2 (33.3)	1 (16.7)	1 (16.7)	1 (16.7)		
Tertiary	9 (52.9)	8 (47.1)	0 (0.0)			7 (41.2)	7 (41.2)	2 (11.8)			1 (5.9)	3 (17.6)	4 (23.5)	3 (17.6)	6 (35.3)		
Post-tertiary	30 (54.5)	21 (38.2)	4 (7.3)			19 (34.5)	13 (23.6)	8 (14.5)			4 (7.5)	8 (15.1)	14 (26.4)	6 (11.3)	21 (39.6)		
Parents’ occupation																	
None	3 (60.0)	2 (40.0)	0 (0.0)	3.528	0.642^f^	4 (80.0)	0 (0.0)	1 (20.0)	13.146	0.153^f^	0 (0.0)	2 (40.0)	1 (20.0)	0 (0.0)	2 (40.0)		
Self-employed	3 (33.3)	5 (55.6)	1 (11.1)			2 (22.2)	4 (44.4)	3 (33.3)			1 (11.1)	1 (11.1)	3 (33.3)	3 (33.3)	1 (11.1)	9.695	0.692^f^
Public servant	21 (61.8)	11 (32.4)	2 (5.9)			13 (38.2)	10 (29.4)	2 (5.9)			4 (11.8)	5 (17.2)	8 (23.5)	4 (11.8)	13 (38.2)		
Private employment	16 (51.6)	14 (45.2)	1 (3.2)			11 (35.5)	8 (25.8)	5 (16.1)			1 (3.4)	5 (17.2)	9 (31.0)	3 (10.3)	11 (37.9)		
Parents’ monthly income																	
None	1 (50.0)	1 (50.0)	0 (0.0)	11.733	0.228^f^	1 (50.0)	0 (0.0)	0 (0.0)	8.279	0.923^ f^	0 (0.0)	0 (0.0)	1 (50.0)	0 (0.0)	1 (50.0)	8.279	0.923^f^
≤£500	3 (37.5)	3 (37.5)	2 (25.0)			3 (37.5)	2 (25.0)	1 (12.5)			1 (12.5)	0 (0.0)	2 (25.0)	2 (25.0)	3 (37.5)		
£501-£1,500	16 (69.6)	7 (30.4)	0 (0.0)			11 (47.8)	9 (39.1)	1 (4.3)			1 (4.3)	4 (17.4)	7 (30.4)	1 (4.3)	10 (43.5)		
£1,501-£2,500	14 (51.9)	11 (40.7)	2 (7.4)			10 (37.0)	9 (33.3)	3 (11.1)			2 (7.7)	5 (19.2)	5 (19.2)	5 (19.2)	9 (34.6)		
≥£2,501	9 (47.4)	10 (52.6)	0 (0.0)			6 (31.6)	2 (10.5)	6 (31.6)			2 (11.1)	3 (16.7)	6 (33.3)	2 (11.1)	5 (27.8)		

**Table 10:** Summary analysis of the association between the parents’ social status and their adequate knowledge of milk fluoridation Data represented as frequency (N) and percentages (%). Statistical association tested using Chi square or Fisher's test (superscript f where applicable). p < 0.05 considered significant.

	Adequate knowledge					Fluoridation benefits oral health of children					Fluoridation prevents dental caries					Milk preferable to other forms of fluoridation				
Variables	Agree	Disagree	Not sure	Chi square	p-value	Agree	Disagree	Not sure	Chi square	p-value	Agree	Disagree	Not sure	Chi square	p-value	Agree	Disagree	Not sure	Chi square	p-value
Parents’ educational level																				
None	0 (0.0)	1 (100.0)	0 (0.0)	6.604	0.635^f^	0 (0.0)	0 (0.0)	1 (100.0)	3.703	0.830^f^	0 (0.0)	0 (0.0)	1 (100.0)	3.434	0.861^f^	0 (0.0)	0 (0.0)	1 (100.0)	10.898	0.062^f^
Primary	0 (0.0)	0 (0.0)	1 (100.0)			0 (0.0)	0 (0.0)	1 (100.0)			0 (0.0)	0 (0.0)	1 (100.0)			0 (0.0)	0 (0.0)	1 (100.0)		
Secondary	2 (40.0)	1 (20.0)	2 (40.0)			3 (60.0)	0 (0.0)	2 (40.0)			2 (40.0)	0 (0.0)	3 (60.0)			3 (60.0)	1 (20.0)	1 (20.0)		
Tertiary	3 (17.6)	6 (35.3)	8 (47.1)			5 (29.4)	1 (5.9)	11 (64.7)			5 (31.3)	2 (12.5)	9 (56.3)			6 (37.5)	3 (18.8)	7 (43.8)		
Post-tertiary	13 (25.5)	10 (19.6)	28 (54.9)			22 (43.1)	3 (5.9)	26 (51.0)			18 (35.3)	2 (3.9)	31 (60.8)			9 (17.6)	4 (7.8)	38 (74.5)		
Parents’ occupation																				
None	0 (0.0)	2 (50.0)	2 (50.0)	6.145	0.478^f^	1 (25.0)	0 (0.0)	3 (75.0)	5.019	0.635^f^	1 (25.0)	0 (0.0)	3 (75.0)	8.922	0.233^f^	2 (50.0)	0 (0.0)	2 (50.0)	4.248	0.575^f^
Self-employed	4 (50.0)	1 (12.5)	3 (37.5)			5 (62.5)	0 (0.0)	3 (37.5)			4 (57.1)	0 (0.0)	3 (42.9)			3 (42.9)	1 (14.3)	3 (42.9)		
Public servant	8 (23.5)	9 (26.5)	17 (50.0)			15 (42.9)	1 (2.9)	19 (54.3)			12 (34.3)	0 (0.0)	23 (65.7)			8 (22.9)	3 (8.6)	24 (68.6)		
Private employment	6 (21.4)	5 (17.9)	17 (60.7)			9 (33.3)	3 (11.1)	15 (55.6)			8 (29.6)	4 (14.8)	15 (55.6)			5 (18.5)	4 (14.8)	18 (66.7)		
Parents’ monthly income																				
None	0 (0.0)	1 (50.0)	1 (50.0)	15.918	0.013^f^	0 (0.0)	0 (0.0)	2 (100.0)	6.273	0.731^f^	0 (0.0)	0 (0.0)	2 (100.0)	6.424	0.626^f^	0 (0.0)	0 (0.0)	2 (100.0)	6.751	0.709^f^
≤£500	1 (16.7)	1 (16.7)	4 (66.7)			1 (20.0)	0 (0.0)	4 (80.0)			1 (20.0)	1 (20.0)	3 (60.0)			2 (40.0)	0 (0.0)	3 (60.0)		
£501-£1,500	6 (30.0)	0 (0.0)	14 (70.0)			9 (42.9)	1 (4.8)	11 (52.4)			8 (38.1)	1 (4.8)	12 (57.1)			6 (28.6)	1 (4.8)	14 (66.7)		
£1,501-£2,500	5 (17.2)	13 (44.8)	11 (37.9)			11 (37.9)	3 (10.3)	15 (51.7)			12 (41.4)	2 (6.9)	15 (51.7)			7 (24.1)	6 (20.7)	16 (55.2)		
≥£2,501	6 (35.3)	3 (17.6)	8 (47.1)			9 (52.9)	0 (0.0)	8 (47.1)			4 (25.0)	0 (0.0)	12 (75.0)			3 (18.8)	1 (6.3)	12 (75.0)		

**Table 11:** Summary analysis of the association between the parents’ social status and their attitude towards milk fluoridation Data represented as frequency (N) and percentages (%). Statistical association tested using Chi square or Fisher's test (superscript f where applicable). p < 0.05 considered significant.

	Give children fluoridated milk				Allow your children to be given fluoridated milk				Milk should be optimally fluoridated in Gateshead				
Variables	Yes	No	Chi square	p-value	Yes	No	Chi square	p-value	Agree	Disagree	Not sure	Chi square	p-value
Parents’ educational level													
None	1 (100.0)	0 (0.0)	0.5	1.000^f^	1 (100.0)	0 (0.0)	5.002	0.299^f^	0 (100.0)	0 (0.0)	1 (100.0)	2.477	0.977^f^
Primary	1 (100.0)	0 (0.0)			0 (0.0)	1 (100.)			0 (0.0)	0 (0.0)	1 (100.0)		
Secondary	4 (80.0)	1 (20.0)			1 (20.0)	4 (80.0)			2 (50.0)	0 (0.0)	2 (50.0)		
Tertiary	13 (81.3)	3 (18.8)			4 (25.0)	12 (75.0)			7 (43.8)	1 (6.3)	8 (50.0)		
Post-tertiary	40 (80.0)	10 (20.0)			22 (44.9)	27 (55.1)			18 (35.3)	4 (7.8)	29 (56.9)		
Parents’ occupation													
None	3 (75.0)	1 (25.0)	0.732	0.851^f^	1 (25.0)	3 (75.0)	1.207	0.800^f^	2 (50.0)	1 (25.0)	1 (25.0)	5.522	0.326^f^
Self-employed	6 (85.7)	1 (14.3)			2 (28.6)	5 (71.4)			3 (50.0)	1 (16.7)	2 (33.3)		
Public servant	27 (77.1)	8 (22.9)			13 (38.2)	21 (61.8)			13 (38.2)	1 (2.9)	20 (58.8)		
Private employment	22 (84.6)	4 (15.4)			12 (46.2)	14 (53.8)			9 (32.1)	2 (7.1)	17 (60.7)		
Parents’ monthly income													
None	2 (100.0)	0 (0.0)	0.832	0.978^f^	2 (100.0)	0 (0.0)	6.624	0.155^f^	0 (0.0)	0 (0.0)	2 (100.0)	0.832	0.978^f^
≤£500	4 (80.0)	1 (20.0)			2 (40.0)	3 (60.0)			3 (50.0)	0 (0.0)	3 (50.0)		
£501-£1,500	16 (76.2)	5 (23.8)			5 (23.8)	16 (76.2)			9 (42.9)	1 (4.8)	11 (52.4)		
£1,501-£2,500	24 (82.8)	5 (17.2)			10 (35.7)	18 (64.3)			11 (39.3)	4 (14.3)	13 (46.4)		
≥£2,501	12 (80.0)	3 (20.0)			8 (53.3)	7 (46.7)			4 (37.5)	0 (0.0)	11 (73.3)		

**Table 12:** Multivariate logistic regression for parents’ educational level as a predictor of parents’ knowledge of milk fluoridation

Parents’ educational level	B	S.E.	Sig.	Exp(B)
None	-19.662	40,192.970	1.000	0.000
Primary	-19.662	40,192.970	1.000	0.000
Secondary	1.946	0.984	0.048	7.000
Tertiary	1.030	0.634	0.104	2.800
Post-tertiary	Reference group			

As illustrated in Table 12, having secondary level education increases the likelihood (p = 0.048) seven times (B = 7.000) of the respondents having adequate knowledge of fluoridation regarding the post-tertiary educational level; however, having no, primary, and tertiary education has no predictive relationship (Table 12). Conversely, parents’ monthly income could not serve as a predictor of their knowledge of milk fluoridation (Table 13).

**Table 13:** Multivariate logistic regression for parents’ monthly income as a predictor of parents’ knowledge of milk fluoridation

Parents’ monthly income	B	S.E.	Sig.	Exp(B)
None	-20.597	28,420.722	0.999	0.000
≤£500	-1.003	1.207	0.406	0.367
£501-£1,500	-0.241	0.704	0.732	0.786
£1,501-£2,500	-0.962	0.707	0.173	0.382
≥£2,501	Reference group			

## Discussion

As of today, only systematic reviews from decades ago remain the primary source of information regarding milk fluoridation [15,16]. However, through its Borrow Foundation, the UK leads the world in research on milk fluoridation, even though the program has only been implemented in a few regions [16].

Milk remains an essential part of children’s diets, providing vital nutrients such as proteins, minerals, and vitamins [17]. It has been successfully used as a vehicle for fluoride delivery, especially in areas without water fluoridation schemes [5]. This method reduces the need for expensive and less accessible treatment-based oral health programs, particularly in rural areas [17].

The demographic data from respondents in this study showed a good distribution, with most respondents in their prime age range (31-45 years). Most children practiced good oral hygiene, brushing at least once or twice a day. Previous studies have shown that daily brushing can reduce the risk of dental caries by 19.5% [2].

In this study, sugar consumption-identified as the primary risk factor for dental caries [18]-was noted to be frequent among children. Sweet snacks consumed at bedtime pose a greater risk, especially when dental visits are infrequent [18]. Excessive sugar intake is linked to tooth decay in children, with social determinants such as income and education playing a significant role in dietary habits [8,19,20]. More than necessary intake of sugar has also been linked with tooth decay in children residing in the US, it was observed that the social determinants of health greatly influenced the habit, and upstream approaches including the outright ban of sugary beverages in schools could promote less sugar intake by children [20]. Similar research [10] reported that the prevalence of oral diseases and the control of sugary snacks play a significant role in children’s oral health.

The role of parents in promoting good oral hygiene was observed to be significant in this study [11]. Parents only know the importance of oral health before they can seek professional help from dentists; however, this may be prevented by several structural obstacles, such as transportation policies and school absence policies [21].

Positive parenting is often associated with lower rates of dental caries, and in this study, most respondents had children with good oral health. However, a significant number of respondents (73%) had no knowledge of milk fluoridation. This finding mirrors similar research in Palestine, where awareness of milk fluoridation was also low [1]. The high prevalence of any health issue has been attributed to poor sociodeterminants of health relating to income, education, and housing [6]. This may be true because low income can prevent the accessibility and utilization of health facilities, as the cost of medication and treatments becomes a secondary option for the less privileged.

It was also observed that parents with secondary education can be used as a predictor of the knowledge of milk fluoridation. Prior studies have reported a direct association between parental education, family income, and the oral health of children [22]. Due to the high number of educated respondents in this study, it was expected that they would have had at least some prior knowledge of fluoridation from the internet, social networking, magazines, and notice boards, but this is not the case. The majority of the people studied had a low socioeconomic status, earning an average income. One interesting revelation is the fact that most of the respondents had a post-tertiary educational level, but only respondents with secondary education had a strong, significant relationship with knowledge of fluoridation. This is quite surprising because it is expected that as the educational level progresses, the knowledge of fluoridation will also increase. This, however, is not the case in our study as the respondents’ monthly income was not significant and, as such, cannot serve as a predictor of their knowledge of milk fluoridation.

Protection motivation theory makes use of persuasive messages. It argues that predicting an individual's intentions is influenced by two main cognitive processes, namely, the threat appraisal and the coping appraisal [12]. It may be that if parents had adequate knowledge about the seriousness of dental caries (perceived severity), they would get to understand that bad oral hygiene is a major risk factor for dental caries (perceived vulnerability). Also, it may be possible that having the right information about milk fluoridation can bring about a preventive response within a community that may avert the perceived threat (response efficacy) of dental caries, but it depends majorly on the individual’s ability to initiate and carry through the process (self-efficacy). The sense of self-efficacy of an individual is independent of barriers [23]. Therefore, a person who feels highly self-empowered might not be able to get through such barriers, which may include the cost of a balanced meal, the cost of going to the dentist, or the timing needed for monitoring their ward's brushing routine, especially in deprived communities. From the public health perspective, when parents are provided with the appropriate knowledge, they are more able to make informed decisions [24]. Thus, enlightening educational programmes and campaigns aimed at motivation and finally behavioural change with regard to milk fluoridation is a need in Gateshead [15].

Most research, particularly that evaluating interventions, has acknowledged that while there are success stories, several limitations have also been reported [25]. These studies indicate the effectiveness of relying on theoretical frameworks; however, depending on the research questions and context, this may also be a bias, even though studies suggest that such theories must be designed based on economic, social, and cultural characteristics in context. In addition, it has been reported that quantitative empirical research ensures validity through rigorous clarification and evaluates instrument validity and reliability through statistical tests [26]. As also reported [26], quantitative research is confirmatory and deductive. However, that seems not to be the case with this current study, as the low response rate was a major limitation, and hence, the results of this study may not be generalized.

The validity of the research instrument is the primary strength of this study; despite this, major limitations were time constraints and selection bias. As this study was cross-sectional and had data collected once, it means the associations between the knowledge and attitudes of parents of young children in Gateshead and their social status (defined by their educational level, monthly income, and occupation) may not be interpreted as causal. Also, selection bias may have resulted from respondents' convenient and voluntary participation, as observed in the over 65% of nationalities being of Black descent. This limitation may have been overcome if the study had been for a longer period, as evident in longitudinal research.

## Conclusions

This study provides valuable insight into the knowledge and attitudes of parents towards milk fluoridation in Gateshead, UK. The findings indicate a significant gap in knowledge about milk fluoridation, which could impact the acceptance of such programs. In order to reduce the prevalence of dental caries, improving oral hygiene practices and implementing a safe community-wide fluoridation scheme, such as milk fluoridation, are essential. The continued distribution of free milk supplements in deprived communities will further support the sustainability of these programs.

Following our conclusions, we recommend that in Gateshead, UK, parental involvement in children’s oral hygiene should be encouraged, with regular dental visits even in the absence of complaints. The Ministry of Health and charitable organisations can enhance parents' knowledge and attitudes regarding milk fluoridation through educational campaigns, pamphlets, and documentaries. Also, the WHO recommends reducing sugar consumption to below 5% of total energy intake. Policies aimed at reducing sugar consumption could help reduce dental caries prevalence. Additionally, the government could sponsor milk fluoridation programs in deprived areas of the UK and offer more than just subsidies. More campaigns should raise awareness of the significance of milk fluoridation in children's oral health, and lastly, more research should be conducted to address the limitations of this study. Future studies should investigate the risk factors associated with childhood dental caries using a mixed-methods approach.

## Appendices

**Table 14:** Research questionnaire

Section A: Demographic data		
S/N	Question	Options
1	Sex (parent)	☐ Male ☐ Female
2	Age (parent)	☐ ≤30 years ☐ 31–45 years ☐ ≥46 years
3	Nationality	☐ Black ☐ Asian or Asian British ☐ Mixed or multiple ethnic groups ☐ White
4	District in Gateshead	☐ Central ☐ East ☐ Inner West ☐ South ☐ West
5	Education	☐ None ☐ Primary ☐ Secondary ☐ Tertiary ☐ Post-tertiary
6	Occupation	☐ None ☐ Self-employed ☐ Public servant ☐ Private employment ☐ Other
7	Monthly income (£)	☐ None ☐ ≤500 ☐ 500–1,500 ☐ 1,500–2,500 ☐ ≥2,500
8	Sex (child)	☐ Male ☐ Female
9	Age (child)	☐ ≤4 years ☐ 5–7 years ☐ 8–10 years ☐ ≥10 years
Section B: Child oral health		
S/N	Question	Options
11	Frequency of toothbrushing	☐ Once daily ☐ At least twice ☐ Once weekly ☐ >Once weekly ☐ Never
12	Frequency of sweet snacks	☐ Once daily ☐ At least twice ☐ Once weekly ☐ >Once weekly ☐ Never
13	Dentist visits	☐ Every term ☐ Once yearly ☐ Twice yearly ☐ >Twice yearly ☐ Never
Section C: Parental perception of child's oral health		
S/N	Question	Options
14	Food caught in teeth	☐ Never ☐ Every day ☐ Weekly ☐ Twice weekly
15	Tooth pain	☐ Never ☐ Every day ☐ Weekly ☐ Twice weekly
16	Difficulty eating hot/cold foods	☐ Never ☐ Every day ☐ Weekly ☐ Monthly
17	Mouth breathing	☐ Never ☐ Every day ☐ Weekly ☐ Monthly
18	Restricted diet	☐ Never ☐ Every day ☐ Weekly ☐ Monthly
19	Worried about appearance	☐ Never ☐ Once/day ☐ ≥Twice/day ☐ Weekly ☐ >Weekly
20	Embarrassed	☐ Never ☐ Once/day ☐ ≥Twice/day ☐ Weekly ☐ >Weekly
21	Anxious	☐ Never ☐ Once/day ☐ ≥Twice/day ☐ Weekly ☐ >Weekly
22	Avoid speaking in class	☐ Never ☐ Once/day ☐ ≥Twice/day ☐ Weekly ☐ >Weekly
23	Avoid talking to children	☐ Never ☐ Once/day ☐ ≥Twice/day ☐ Weekly ☐ >Weekly
24	Left out by children	☐ Never ☐ Every day ☐ Weekly
Section D: Knowledge & opinions of milk fluoridation		
S/N	Question	Options
25	Knowledge about milk fluoridation	☐ Agree ☐ Disagree ☐ Not sure
26	Effective prevention methods	☐ Water fluoridation ☐ Milk fluoridation ☐ Topical fluoride ☐ Toothpastes ☐ Supplements ☐ Rinses ☐ Caries cannot be prevented
27	Milk fluoridation benefits children	☐ Agree ☐ Disagree ☐ Not sure
28	Milk fluoridation prevents caries	☐ Agree ☐ Disagree ☐ Not sure
29	Prefer milk fluoridation	☐ Agree ☐ Disagree ☐ Not sure
30	Give child fluoridated milk	☐ Yes ☐ No
31	Allow fluoridated milk	☐ Yes ☐ No
32	School milk is fluoridated	☐ Agree ☐ Disagree ☐ Not sure
33	Support optimal fluoridation	☐ Agree ☐ Disagree ☐ Not sure
34	Reasons against fluoridation	☐ Environmental hazard ☐ Violates freedom ☐ Health effects ☐ Allergies ☐ Bone issues ☐ Fluorosis ☐ Cancer ☐ Neurological ☐ Costly
